# The internal state of medium spiny neurons varies in response to different input signals

**DOI:** 10.1186/1752-0509-4-26

**Published:** 2010-03-17

**Authors:** Zhen Qi, Gary W Miller, Eberhard O Voit

**Affiliations:** 1 Department of Biomedical Engineering, Georgia Institute of Technology and Emory University Medical School, Atlanta, GA 30332, USA; 2 Center for Neurodegenerative Disease, Emory University School of Medicine, Atlanta, GA 30322, USA; 3 Integrative BioSystems Institute, Georgia Institute of Technology, Atlanta, GA 30332, USA; 4 Department of Environmental and Occupational Health, Rollins School of Public Health, Emory University, Atlanta, GA 30322, USA

## Abstract

**Background:**

Parkinson's disease, schizophrenia, Huntington's chorea and drug addiction are manifestations of malfunctioning neurons within the striatum region at the base of the human forebrain. A key component of these neurons is the protein DARPP-32, which receives and processes various types of dopamine and glutamate inputs and translates them into specific biochemical, cellular, physiological, and behavioral responses. DARPP-32's unique capacity of faithfully converting distinct neurotransmitter signals into appropriate responses is achieved through a complex phosphorylation-dephosphorylation system that evades intuition and predictability.

**Results:**

To gain deeper insights into the functioning of the DARPP-32 signal transduction system, we developed a dynamic model that is robust and consistent with available clinical, pharmacological, and biological observations. Upon validation, the model was first used to explore how different input signal scenarios are processed by DARPP-32 and translated into distinct static and dynamic responses. Secondly, a comprehensive perturbation analysis identified the specific role of each component on the system's signal transduction ability.

**Conclusions:**

Our study investigated the effects of various patterns of neurotransmission on signal integration and interpretation by DARPP-32 and showed that the DARPP-32 system has the capability of discerning surprisingly many neurotransmission scenarios. We also screened out potential mechanisms underlying this capability of the DARPP-32 system. This type of insight deepens our understanding of neuronal signal transduction in normal medium spiny neurons, sheds light on neurological disorders associated with the striatum, and might aid the search for intervention targets in neurological diseases and drug addiction.

## Background

The basal ganglia form a functional complex of neurons that are located at the base of the human forebrain. Malfunctioning of this complex has been associated with Parkinson's disease, schizophrenia, Huntington's chorea, and drug addiction [1]. The basal ganglia system receives most of its input through the striatum, which consists primarily of medium spiny neurons. These neurons are equipped with a protein, called DARPP-32 (dopamine- and cAMP-regulated phosphoprotein with 32 kDa molecular weight), that is the key mediator of signal processing [2]. In response to dopamine signals, DARPP-32 mediates a variety of biochemical, cellular, and physiological effects [3,4], including changes in the ion channel state of membranes, altered transcription factor activity, up- or down-regulation of gene expression, and reward [5,6]. Furthermore, DARPP-32 has important functions in behavior, such as changes in motor activity. In addition to dopamine signals, DARPP-32 processes excitatory glutamatergic signals from the cortex, which are similarly crucial inputs to the striatum [7,8]. Thus, DARPP-32 serves as a centralized location for receiving, integrating and processing signals from several neurotransmitters and constitutes a key element in the important cortico-striato-pallido-thalamo-cortical loop.

**Figure 1:**
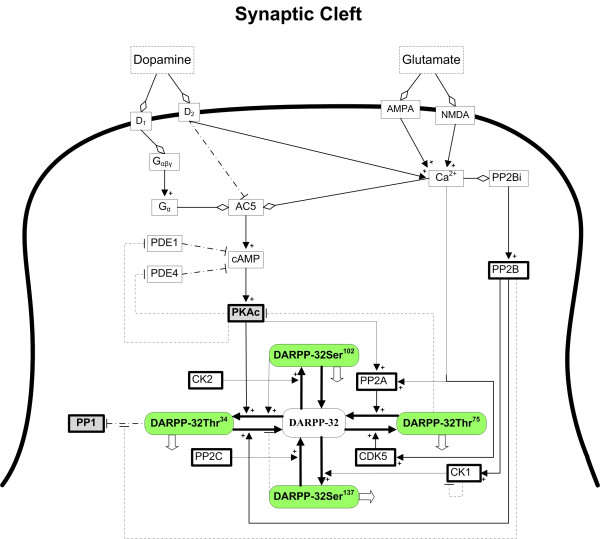
**Signal integration and interpretation by the DARPP-32 system in dendrites of medium spiny neurons in the striatum**. The membrane of spiny neurons (solid curved line) contains receptors for the neurotransmitters dopamine and glutamate, which had been released by other neurons into the synaptic cleft. Diamonds show molecular binding, while arrows with plus signs designate activation or enzymatic reactions and dash-dotted lines with bars indicate inhibition. Shaded boxes in green color represent phosphorylated forms of DARPP-32, with labels indicating the specific phosphorylated site, and arrows showing possible transitions between the different phosphorylation states. Open block arrows indicate possible multiple-site phosphorylation. Please refer to the Figure 2 for a detailed map of all possible transitions between different phosphorylation forms. Abbreviations are: dopamine receptor of D_1 _subtype (D_1_), dopamine receptor of D_2 _subtype (D_2_), G protein and subunits (G_αβγ_, G_α_), adenylate cyclase (AC5), cyclic AMP (cAMP), phosphodiesterases (PDE1, PDE4), protein kinase A (PKA, PKAc), alpha-amino-3-hydroxy-5-methyl-4-isoxazolepropionic acid (AMPA), N-methyl-D-aspartate (NMDA), protein phosphatase 2B (PP2Bi, PP2B), protein phosphatase-1 (PP1), protein phosphatase 2A (PP2A), cyclin-dependent kinase 5 (CDK5), casein kinase 2 (CK2), casein kinase 1 (CK1), protein phosphatase 2C (PP2C), dopamine- and cAMP-regulated phosphoprotein with 32 kDa molecular weight (DARPP-32). Phosphorylation of DARPP-32 is indicated by the corresponding amino acid and location, such as DARPP-32Thr^34^. See Figure 2 for multiple-site phosphorylation.

**Figure 2:**
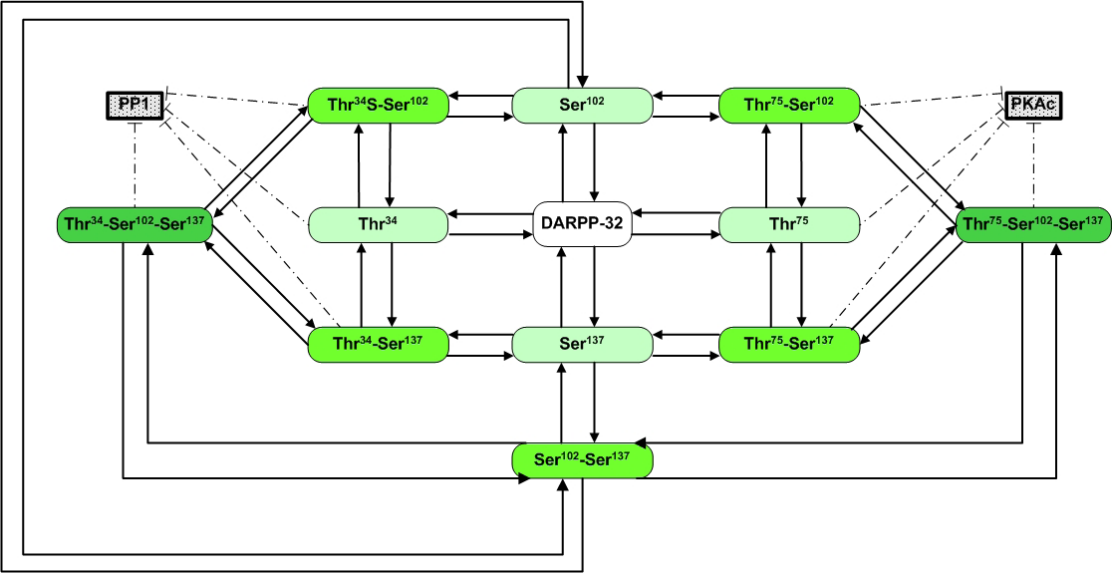
**Phosphorylation states of DARPP-32**. Differently shaded boxes show singly, doubly and triply phosphorylated states of DARPP-32, and labels indicate phosphorylation positions. Arrows indicate transitions between different phosphorylation forms through enzymatic reactions. Dash-dotted lines with bars represent inhibition. Abbreviations are: protein phosphatase-1 (PP1), protein kinase A (PKAc), dopamine- and cAMP-regulated phosphoprotein with 32 kDa molecular weight (DARPP-32). Different phosphorylation states of DARPP-32 are indicated by the corresponding amino acid(s) and location(s). For instance, (Thr^34^-Ser^102^) refers to DARPP-32 phosphorylated simultaneously at positions 34 and 102.

Experimental evidence has shown that DARPP-32 functions by translating the incoming neuronal signals into distinct activity profiles of kinases and phosphatases. This translation process is based on phosphorylation and dephosphorylation at multiple sites of DARPP-32 and accomplished through a complicated signal transduction network that uses a second messenger system and a biochemical pathway activated by calcium (Figures 1 and 2). Specifically, when dopamine binds to its postsynaptic receptor of D1 subtype, a secondary messenger system is trigged that leads to the production of cyclic AMP (cAMP). This messenger molecule in turn activates protein kinase A (PKA). Both cAMP and PKA are critically regulated by phosphodiesterase (PDE), which has multiple isoforms such as PDE1 and PDE4 [9]. PKA then phosphorylates DARPP-32 at a specific threonine residue (Thr34) and thereby converts it into a potent inhibitor of protein phosphatase-1 (PP1) [2,10]. In contrast to dopamine, glutamate binds to its own ionotropic receptors (*e.g*., AMPA and NMDA), which causes Ca^2+ ^cations to flow into the cell. This induced Ca^2+ ^influx reduces the inhibition of PP1 by activating protein phosphatase 2B (PP2B). PP2B dephosphorylates DARPP-32 at the threonine site and enhances the inhibition of PKA by increasing phosphorylation of DARPP-32 at another threonine residue, which is phosphorylated by cyclin-dependent kinase 5 (CDK5) [11-13].

The brief description of event chains indicates that the DARPP-32 signal transduction system balances the effects of competing triggers, which themselves are dynamic and depend on their recent histories. As a consequence, the integration and interpretation of neuronal signals by DARPP-32 can vary in response to different trains of input signals. In other words, different signal patterns, such as a single signal *vs*. coexistent signals, a one-time pulse *vs*. repetitive pulses, or signals for short-term *vs*. long-term plasticity, can have distinct effects on rewards, learning, memory, locomotion and/or synaptic plasticity.

The multitude of graded effects and interactions of the complex DARPP-32 system easily evades intuition and would benefit from a representation and analysis with methods of systems biology. Such an analysis could yield answers to questions regarding the specifics of the operation and design of the signal transduction system. For instance, one might ask which mechanisms are dominant in a given signaling situation and within a specific context. Do these mechanisms function synergistically or antagonistically? To what degree do they contribute to different modes of signal integration that are required by relevant brain functions or implicated in neurological diseases?

The goal of this work is to investigate such questions systematically and quantitatively with methods of computational systems biology. At the core of our approach is a dynamic model that is capable of accounting for the many components and processes associated with the DARPP-32 system in a numerical fashion. The details of this model are described in the *Methods *Section, and the model itself is presented explicitly and completely in Additional file 1. Upon formulating, testing, and validating the model, we simulated various patterns of neurotransmission and studied their effects on signal integration and interpretation by DARPP-32. The simulations and analyses addressed both the steady-state and the dynamics of the system. Moreover, systematic perturbations with the validated model revealed which mechanisms are dominant in specific signal input situations. This type of insight deepens our understanding of neuronal signal transduction in normal medium spiny neurons, could possibly shed light on mechanisms of neurological disorders associated with the striatum, and might aid the search for intervention targets in neurological diseases.

## Results

### Validation of the model

The model, as detailed in the *Methods *Section, serves as an exploratory platform for scenario simulations and their evaluations. However, before the model can be used in this capacity, it must be tested and validated against available experimental and clinical data on the biochemistry, pharmacology, and electrophysiology of the system. The available experimental observations cover both static and dynamic behaviors of the DARPP-32 system. Most of them were carried out on rats.

Under normal, unstimulated base conditions, the degree of DARPP-32 phosphorylation at Thr34 and Thr75 was experimentally determined as 0.5-1.0% and 26%, respectively [14-16]. Our model shows phosphorylation rates of ~0.7% and ~28.6% of total DARPP-32 molecules, respectively. The model also suggests that phosphorylation at Ser102 and Ser137, as well as unphosphorylated DARPP-32 account for ~3.0%, ~28.7%, and ~48.9%, respectively. Meanwhile, active PKA and PP1 show levels of ~0.7 nM and ~4.7 μM, respectively.

In laboratory experiments, the Thr34 phosphorylation level rose over 6-fold within 2 to 10 minutes when the dopamine signal was activated following a stimulus [17-19], while Thr75 decreased to about half its base value [16]. Quite consistently, the model shows that Thr34 increases about 7-fold within 3 minutes and Thr75 decreases by ~35% following a 5-minute stimulation of 10 μM dopamine. In experiments where dopamine was depleted from the striatum, Thr75 (but not Thr34) rose substantially [20], and again this observation is reproduced well in a model simulation.

Similarly, the effects of stimulatory glutamate signals were assessed. Experiments indicated that an enhanced or prolonged glutamate signal increases the Ca^2+ ^concentration, which in turn leads to a rapid 50% reduction in the levels of Thr34 and Thr75 [21,22]. In the corresponding model simulation of a 5-minute stimulation with 1 μM Ca^2+^, Thr34 and Thr75 phosphorylation is reduced to ~30% and ~55% of untreated levels within 1 minute. When the stimulus ceases, it takes about 6 minutes for Thr75 and 20 minutes for Thr34 to return to their basal levels [21,22], and again these simulation results are consistent with experimental observations.

The dopamine and glutamate signals modulate each other's effects on DARPP-32. Using concurrent signals, the model shows that glutamate inhibits a Thr34 increase and enhances a Thr75 decrease induced by the dopamine signal, whereas dopamine counteracts a Thr34 decrease caused by glutamate and raises its level. Again, these simulation results are consistent with experimental findings [17].

Genetic and pharmacological interventions to the DARPP-32 system were also simulated with the model and the results were compared with experimental data. The effect of an intervention is simulated most readily by adjusting the numerical configuration of the model correspondingly. For example, 90% inhibition of PP2B by cyclosporin A is implemented as a 90% reduction in PP2B enzyme activity since the cyclosporin A is not in the model. Experiments showed that CDK5 inhibition by 10 μM roscovitine reduced Thr75 by ~73% and raised Thr34 about 10 fold [16,19,23,24]. Consistently, a model simulation of CDK5 inhibition showed that Thr75 decreases by ~60%, while Thr34 increases more than 7 fold.

Other experiments showed that CDK5 activation by up-regulation of its activator p35 attenuates the effects of cocaine-mediated dopamine transmission on raising Thr34 and reducing Thr75 [25]. This observation is also well reproduced by a model simulation. Furthermore, manipulations on some other important kinases and phosphatases are consistent with experimental observations. For instance, the model shows that 90% inhibition of PP2B by cyclosporin A eliminates the effect of Ca^2+ ^on Thr34 phosphorylation [17], and that almost complete inhibition of PP2A by 1 μM okadaic acid raises the Thr75 level by over 3 fold [19,22,26].

### Effects of different signals

After the model has been tested and proved to have the ability to reproduce diverse experimental observations, it can be utilized to study the characteristics and capabilities of the DARPP-32 system in integrating and interpreting different types of neuronal signals, as described in the *Methods *Section. The main goal of this analysis was to answer the question of how the DARPP-32 system manages to map different input scenarios reliably into distinct responses. In this section, we analyze the effects of different inputs on the state of the DARPP-32 system, while the following section will shed light on the internal mechanisms that implement this mapping. These modeling activities generate actual predictions since corresponding experiments have not yet been documented in the literature.

**Figure 3:**
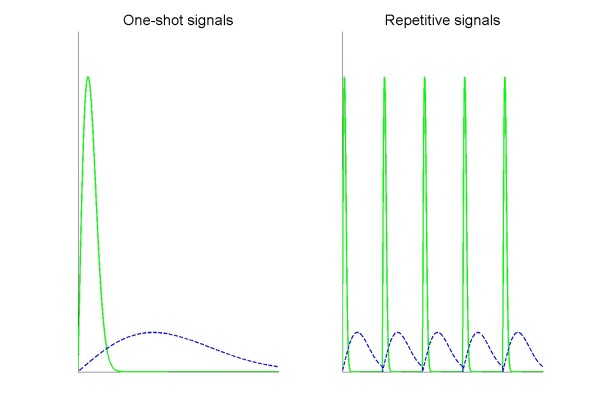
**Schematic representation of input signal patterns**. The left panel illustrates one-shot scenarios of input signals, while the right panel shows repetitive input signals. Solid lines represent transient signals, while dashed lines show sustained input signals.

Relevant signals included dopamine and glutamate stimulation, and they were implemented by specifically resetting the extracellular dopamine level and the intracellular concentration of Ca^2+^, respectively. As discussed in detail in the *Methods *Section, we tested different input signal types, namely: individual or combined dopamine and glutamate signals; transient or sustained signals; one-shot pulse or repetitive pulse signals; signals of varying time scale; and signals of high or low amplitude. To configure these input signals quantitatively, we first assessed which time scales were most realistic. According to experimental measurements, the dopamine release stimulated by action potentials at different frequencies can have a time scale of between milliseconds (< 2 Hz stimuli) and seconds (~20 Hz stimuli) [27]. The Ca^2+ ^current responds to a glutamate signal as fast as milliseconds [28]. Accordingly, we set time scales of dopamine and calcium signals between milliseconds and seconds. In accordance with the frequencies of different brain waves, we set the input signals such that a sustained input has a 5-fold larger width than a transient input, and that a repetitive input comprises five pulses instead of one pulse in a one-shot input (Figure 3).

#### Dopamine signals

**Figure 4:**
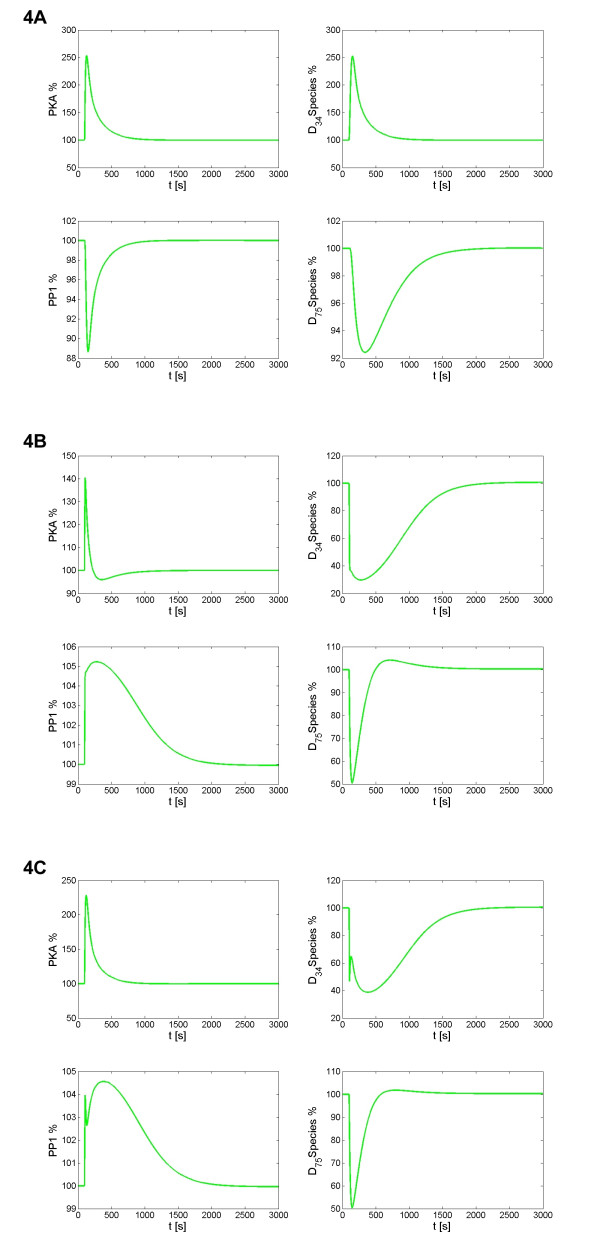
**Typical responses of the DARPP-32 system to dopamine and glutamate (Ca^2+ ^influx) signals**. Panel A shows a typical response of the system to a sole dopamine signal, while panel B exhibits a representative response of the system to a sole glutamate (Ca^2+ ^influx) signal. Panel C shows a common response of the system to a combination of dopamine and Ca^2+ ^signals.

When the DARPP-32 system was stimulated with different patterns of dopamine signals, its responses were typically characterized by a spike in PKA and D_34 _* (DARPP-32 phosphorylated at Thr34), as well as a trough in PP1 and D_75 _* (DARPP-32 phosphorylated at Thr75) (Figure 4A). The amplitudes of these spikes and troughs depended on duration, intensity, and shape of a signal.

#### Glutamate signals

Glutamate signals are represented in the model as changes in Ca^2+ ^influx. In response to most types of Ca^2+ ^influx, the DARPP-32 system commonly responded with spikes in both PKA and PP1, which were accompanied by troughs in D_34 _* and D_75 _* (Figure 4B). Compared to responses to dopamine signals, PKA exhibited an extra undershoot and consequently went through a refractory period, while D_75 _* showed an extra overshoot and a subsequent refractory period. The main distinctive feature between the effects of dopamine and those of glutamate is the qualitative difference in the dynamics of PP1 and D_34 _*.

#### Combined signals

When the system was exposed to simultaneous dopamine and glutamate signals, the DARPP-32 system responses often resembled the responses to Ca^2+ ^signals alone (Figure 4C). However, the undershoot in PKA and its refractory period were no longer seen, and the overshoot in D_75 _* was less obvious or disappeared. The amplitude of the spike in PKA was determined mainly by the dopamine, while the amplitude of the trough in D_75 _* was mostly attributed to the Ca^2+ ^flux.

#### Types of signals

Instead of inducing typical responses as described above, some signal patterns qualitatively altered the steady state of the DARPP-32 system or caused unusual dynamic behaviors. To code the characteristics of a signal pattern in a succinct notation, we use in the following the notation [*signal time scale *(milliseconds or seconds)/*signal number *(one-shot or repetitive)/*signal shape *(transient or sustained)/*signal amplitude *(high or low amplitude)].

#### Transient vs. sustained

**Figure 5:**
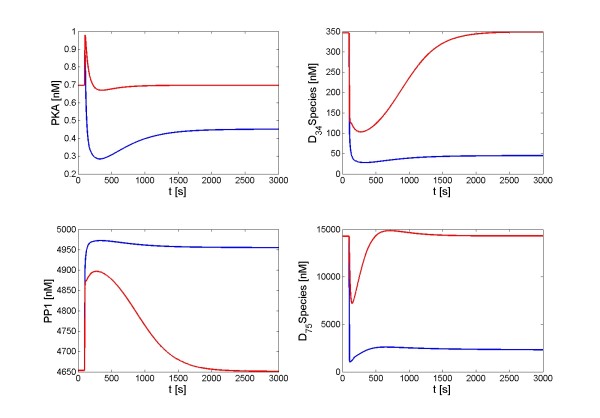
**Different responses of the DARPP-32 system to a transient or a sustained signal**. Input signals are repetitive, high-amplitude Ca^2+ ^influxes (corresponding to glutamate stimulation) with a time scale of milliseconds. Blue lines: Responses to a transient signal; red lines: Responses to a sustained signal.

A transient Ca^2+ ^signal [milliseconds/repetitive/transient/high amplitude] had the capability of switching the DARPP-32 system to a new steady state, which was characterized by elevated PP1 and reduced levels of PKA, D_34 _*, and D_75 _*. This switch should be seen in contrast to the complete recovery of the steady state when the corresponding sustained input signal was applied (Figure 5).

#### One-shot vs. repetitive

**Figure 6:**
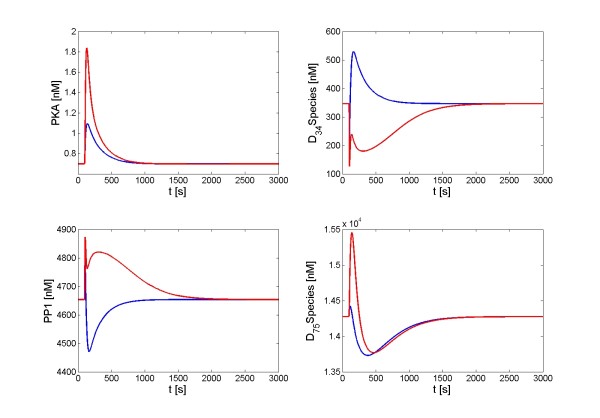
**Different responses of the DARPP-32 system to a one-shot or a repetitive signal**. Input signals are combinations of a dopamine signal and Ca^2+ ^influx. They have low amplitude and a sustained shape with a time scale of seconds. Blue lines: Responses to a one-shot signal; red lines: Responses to a repetitive signal.

A one-shot pulse can induce distinctly different responses of the DARPP-32 system than the corresponding repetitive signal. Figure 6 shows such an example in the case of a combined signal of dopamine and Ca^2+^: A single pulse (dopamine combined with Ca^2+^, seconds/one-shot/sustained/low amplitude) caused a trough in PP1, which is in stark contrast to a spike if the input was a repetitive signal. Also, the one-shot signal induced a spike in D_34 _*, while the repetitive pulse caused a trough. In addition, the spike in D_75 _* was almost erased in the one-shot scenario.

#### High vs. low amplitude

**Figure 7:**
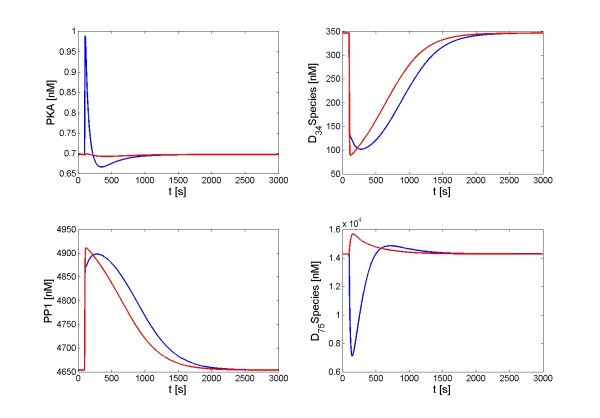
**Different dynamics of the DARPP-32 system in response to a high- or a low- amplitude signal**. Input signals are repetitive, sustained Ca^2+ ^influxes with a time scale of seconds. Blue lines: Responses to a high amplitude signal; red lines: Responses to a low amplitude signal.

**Figure 8:**
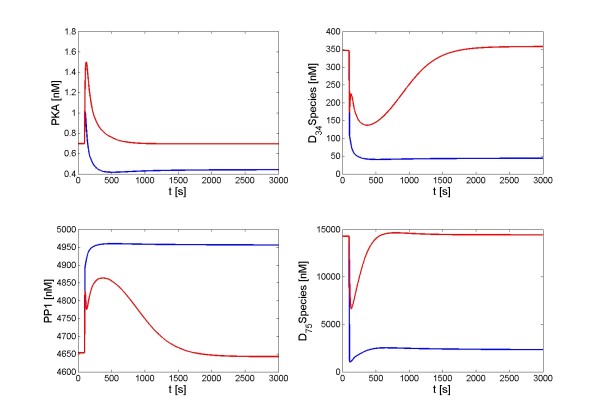
**Different steady states of the DARPP-32 system in response to a high- or a low- amplitude signal**. Input signals are combinations of dopamine stimulation and Ca^2+ ^influx. They are repetitive, transient with a time scale of milliseconds. Blue lines: Responses to a high amplitude signal; red lines: Responses to a low amplitude signal.

A change in signal amplitude can qualitatively alter the dynamic responses of the DARPP-32 system, as it is shown in Figure 7. A Ca^2+ ^signal with high amplitude (seconds/repetitive/sustained/high amplitude) induced a spike and a refractory period in PKA, while the corresponding signal of low amplitude had almost no effect on PKA. The high amplitude signal caused a trough in D_75 _*, while the low amplitude signal had the opposite effect. An input signal of high amplitude could also have a different effect on the steady state of the DARPP-32 system than the corresponding signal of low amplitude. Figure 8 shows that a combined signal of dopamine and Ca^2+ ^(milliseconds/repetitive/transient/high amplitude) alters the steady state of the DARPP-32 system while the counterpart signal of low amplitude does not. The new steady state shown in the high amplitude scenario has an elevated level of PP1 and reduced level of PKA, D_34 _*, and D_75 _*. The time scale is also important; however, its effects could be represented by variations in amplitude and the number of pulses of a signal.

Summarizing this section we can state that different patterns of input signals are clearly differentiated by the DARPP-32 signal transduction system and that they are reflected in the response state of the system (*cf*. Table 1). The next section investigates the robustness of the stimulus-response pattern implementation against perturbations of its internal mechanisms.

**Table 1:** Mapping capability of the DARPP-32 system between neuronal signals and phosphorylation profiles of PKA and PP1

Input Signal		DARPP-32 Status		Output	
**Dopamine^a^**	**Glutamate^a^**	**Thr_34_^b^**	**Thr_75_^b^75**	**PKA^b^**	**PP1^b^**
Any signal of millisecond to second scale		Δ≡	∇≡	Δ≡	∇≡
	ms-sp-tt-ha	∇↓	∇↓	∇↓	Δ↑
	ms-sp-tt-la	∇≡	∇≡	Δ≡	Δ≡
	ms-sp-st-ha	∇≡	∇≡	Δ≡	Δ≡
	ms-sp-st-la	∇≡	∇≡	↔≡	Δ≡
	ms-rp-tt-ha	∇↓	∇↓	∇↓	Δ↑
	ms-rp-tt-la	∇≡	∇≡	Δ≡	Δ≡
	ms-rp-st-ha/la	∇≡	∇≡	Δ≡	Δ≡
	s-sp-tt-ha	∇≡	∇≡	Δ≡	Δ≡
	s-sp-tt-la	∇≡	∇≡	↔≡	↔≡
	s-sp-st-ha	∇≡	∇≡	Δ≡	Δ≡
	s-sp-st-la	∇≡	↔≡	↔≡	Δ≡
	s-rp-tt-ha/la	∇≡	∇≡	Δ≡	Δ≡
	s-rp-st-ha	∇≡	∇≡	Δ≡	Δ≡
	s-rp-st-la	∇≡	Δ≡	↔≡	Δ≡
ms-sp-tt-ha	ms-sp-tt-ha	∇↓	∇↓	∇↓	Δ↑
ms-sp-tt-la	ms-sp-tt-la	∇≡	∇≡	Δ≡	Δ≡
ms-sp-st-ha/la	ms-sp-st-ha/la	∇≡	∇≡	Δ≡	Δ≡
ms-rp-tt-ha	ms-rp-tt-ha	∇↓	∇↓	∇↓	Δ↑
ms-rp-tt-la	ms-rp-tt-la	∇≡	∇≡	Δ≡	Δ≡
ms-rp-st-ha/la	ms-rp-st-ha/la	∇≡	∇≡	Δ≡	Δ≡
s-sp-tt-ha	s-sp-tt-ha	∇≡	∇≡	Δ≡	Δ≡
s-sp-tt-la	s-sp-tt-la	Δ≡	∇≡	Δ≡	∇≡
s-sp-st-ha/la	s-sp-st-ha/la	Δ≡	∇≡	Δ≡	∇≡
s-rp-tt-ha/la	s-rp-tt-ha/la	∇≡	∇≡	Δ≡	Δ≡
s-rp-st-ha/la	s-rp-st-ha/la	∇≡	∇≡	Δ≡	Δ≡

^a^: Abbreviations for neurotransmission patterns are: millisecond (ms), second (s), a single pulse (sp), repetitive pulses (rp), transient transmission (tt), sustained transmission (st), high amplitude signal (ha), and low amplitude signal (la).

^b^: The responses of the DARPP-32 state and its output are presented as *transient dynamics *and *long-term state*. The transient dynamics is described as *trough *(∇), *essentially flat *(↔, and *peak *(Δ) reflecting the main characteristic of the response. The long-term state of phosphorylation is given in relation to the resting level as *decreased *(↓), *basal *(≡), and *increased *(↑).

### Robustness of signal-response implementation in the DARPP-32 system

The qualitative differences seen in the static or dynamic behaviors of the DARPP-32 system in response to different signal patterns raise an interesting question, namely: What are the governing mechanisms responsible for these input-output differences and how robust are they? It is clear that the distinct system responses are critical for the proper functioning of the DARPP-32 signaling network, and that a better elucidation of their mechanistic implementation and robustness could possibly help deepen our understanding of its functioning, identify weak points in the system that are associated with disease outcomes, and provide targets for therapeutic interventions. Thus, in this section we employ systematic perturbations to analyze mechanisms that connect specific signals with the corresponding responses.

In order to assess the robustness of DARPP-32 mediated signal transduction, we simulated perturbations of all processes within the DARPP-32 system one by one to discover the most critical processes. We used signal configurations shown in Figures 5, 6, 7, and 8 as signal scenarios and in the following describe their implementation under the categories *signal shape scenario*, *signal number scenario*, *signal amplitude scenario - dynamics*, and *signal amplitude scenario - steady state*. For each scenario, each process within the DARPP-32 system was separately accelerated or decelerated (including complete blockage) to simulate effects of chemical or genetic perturbations. The effect of each perturbation was assessed, thereby revealing the dominant processes of the signal-response implementation in each scenario.

#### Signal shape scenario (transient vs. sustained)

The most important result of this line of simulations affirms the extraordinary robustness of the DARPP-32 system. We systematically altered every process within the system within a range of 10-fold acceleration or deceleration and found that no single process was so critical that such an alteration in its flux would eliminate the system's ability to discern between a transient and a sustained Ca^2+ ^signal; the appropriate steady states of PKA and PP1 were achieved in all cases. Even under complete inhibition of a process, the DARPP-32 system usually achieved a different steady state when it was subjected to different Ca^2+ ^signals.

However, there are crucial processes that can destroy the proper response pattern when sufficiently inhibited. Not surprisingly, processes transducing the Ca^2+ ^signal are very important. Especially, blockade of activation of PP2B by the Ca^2+ ^signal can eliminate the differences in steady states and thereby prevent the system from distinguishing these signals and responding appropriately. Although these inputs are associated with Ca^2+ ^signals, which correspond to glutamatergic stimulation, the dopamine signal can have a crucial function in this context. Specifically, inhibition of processes for cAMP and PKA production can equalize responses induced by transient and sustained Ca^2+ ^signals and thereby compromise signal discernibility. Because of the important roles of the dopamine pathway and PKA, the inhibition of PKA by D_75 _phosphorylation also contributes substantially to variations in the steady state of the DARPP-32 system.

#### Signal number scenario (one-shot vs. repetitive signal)

Simulations screening for critical processes in this category suggest that the dopamine pathway is crucial for dynamic differences, especially the production processes of cAMP and PKA. Phosphodiesterases, which inhibit the formation of cAMP, similarly make an important contribution to these differences. The Ca^2+ ^pathway, and specifically its activation of PP2B, is also critical for discerning a one-shot from a repetitive signal.

#### Signal amplitude scenario (high vs. low amplitude)

Finally, the critical processes for mediating different signal amplitudes and transducing them to distinct dynamic transients and steady states of the DARPP-32 system were investigated. The dopamine pathway and Ca^2+ ^were both found to be significant contributors. However, the activation of PP2B was no longer identified as crucial. Instead, Ca^2+ ^activation of the phosphatase PP2A and the interaction between PKA and PP2A are dominant for both dynamic and static responses to different signal amplitudes. Although phosphodiesterases can inhibit the formation of cAMP and thus reduce the activity of PKA, they contribute only negligibly to the observed differences in response to high and low signal amplitudes.

## Discussion

The DARPP-32 system integrates different types of dopamine and glutamate signals and transduces them toward appropriate biochemical, cellular, physiological, and behavioral responses, such as motor behavior [29]. It mediates these crucial functions of neurotransmitters through regulating the phosphorylation profiles of PKA and PP1 in an appropriate and adaptive fashion [3,30]. For instance, by means of phosphorylation or dephosphorylation of the glutamate receptors, an incoming neuronal signal can induce changes in the conductance and/or density of these receptors, thereby changing the cell's responsiveness adaptively [31-33]. Also, N/P-type Ca^2+ ^channels and neuronal Na^+^, K^+^-ATPase can be regulated by neurotransmitters via the DARPP-32 system [5]. Furthermore, experiments of DARPP-32 knockout mice demonstrated that DARPP-32 contributes to synaptic plasticity such as long-term depression and long-term potentiation [34]. These long-term forms of synaptic plasticity can be induced by altering gene transcription for synaptic function through PKA and PP1 regulation of transcription factors such as CREB and of immediate early genes such as C-fos and ΔfosB [5,35-37]. In addition, locomotor activity and reward behavior can be regulated through the DARPP-32 system.

Thus, a vast array of studies has confirmed that the DARPP-32 system is a critical mediator for complex neuronal signal transduction. Combining this documented function with our detailed model analyses showing how various patterns of neurotransmission have distinct effects on the internal state of the DARPP-32 system raises an important question, namely: Are the different biochemical states and transient responses of DARPP-32 physiologically and behaviorally significant and could they even correspond to different phenotypes associated with striatal function and dysfunction? Complete answers cannot yet be given and will require further exploration and discovery through cell and animal experiments. A biological phenomenon of relevance in this category is reinforcement learning, with which medium spiny neurons in the striatum can fire more easily under sole cortical input. This functionality is achieved through phosphorylation of AMPA receptors, which can be phosphorylated and dephosphorylated at Ser845 of its GluR1 subunit by PKA and PP1, respectively. This type of phosphorylation and dephosphorylation activity corresponds to the insertion and removal of membrane associated AMPA receptors, respectively [38,39]. Since our model suggests that different input signals can lead to distinct PKA and PP1 activity profiles, it might be worthwhile exploring the connections of these simulated signals with reinforcement learning.

Systematic simulations of local perturbations affecting the DARPP-32 system demonstrated that the system is very robust in its interpretation of the relevant spectrum of neuronal signals. Even under conditions of 10-fold acceleration or deceleration of any single process, the DARPP-32 system was still able to translate input signals into their correct responses. However, the system also contains critical components whose perturbation could interrupt normal behaviors. Among these are mechanisms transducing the Ca^2+ ^signal, especially the activation of PP2B. If this activation is impeded, the dynamics and/or static states of the system may no longer reflect the correct input signal.

The model demonstrates in what manner the dopamine pathway plays an indispensable role in signal interpretation. The primary mechanism is the regulation of the kinase PKA through the production of cAMP and the phosphorylation level of DARPP-32 at the Thr75 site. Depending on input signal patterns, dopamine and glutamate can have synergistic or antagonistic interactions, which contribute to distinctly different responses.

The work presented here focuses primarily on the neurotransmitters dopamine and glutamate. For medium spiny neurons which receive innervation from excitatory glutamatergic neurons and modulatory dopaminergic neurons, our results showed that these two neurotransmitters interact differently within the DARPP-32 system in response to various input signal patterns. Meanwhile, different transmitter systems contribute most to observed discerning capability of different signal patterns. Thus, if an intervention is designed to manipulate signal discernibility by the DARPP-32 system, the choice of the targeted neurotransmitter system has to be based on the specific goal. For such a task, our model can be used to screen the target and simulate the effects of the intentional intervention.

It is known that medium spiny neurons also receive inputs from other neurotransmitters such as serotonin, and it is reasonable to expect that these signals also contribute significantly to functions of medium spiny neurons. These contributions will be the subject of future research. It might also be useful to expand the signal patterns proposed here. For instance, long-term behaviors lasting minutes, hours, or even longer are clearly biologically significant and should be studied with the proposed model as well. Finally, all neurotransmitter signals are controlled by action potentials, ion fluxes, and metabolic pathways in presynapses, where the neurotransmitters are synthesized, degraded, compartmentalized, released, and taken back into a synaptic terminal [40]. Thus, presynapse and postsynapse models should be linked in order to account for a more comprehensive understanding of normal function and dysfunction of the striatum.

## Conclusions

In this study, we investigated systematically and quantitatively the capability of a signal transduction system (the DARPP-32 system) in neuronal signals integration and interpretation with methods of computational systems biology. As shown by our results, the DARPP-32 system implements a sophisticated many-to-many mapping that senses various signal characteristics and their combinations in terms of their biochemical source, shape, amplitude, repetition, and time scale and converts these into distinct internal states. These states correspond initially to distinct phosphorylation profiles of the DARPP-32 molecule and are subsequently translated into different dynamic and static activity configurations of the kinase PKA and the phosphatase PP1.

The amplitude of a signal is a direct function of the intensity of neurotransmitter release and contributes to synaptic plasticity. According to our results, different dopamine and glutamate signal amplitudes can cause the DARPP-32 to switch among its different steady states and/or to change its dynamic responses qualitatively. Similarly, neurotransmissions of different shapes and repetition characteristics can be translated through DARPP-32 into different steady states and/or distinct dynamic responses. Such dicernibility might have important implications in physiological and behavioral phenomena, such as synaptic plasticity.

The discernibility of the DARPP-32 system between various neurotransmission patterns is associated by our systemic analyses with mechanisms transducing the Ca^2+ ^signal, especially the activation of PP2B, and mechanisms regulating the kinase PKA through the production of cAMP and the phosphorylation level of DARPP-32 at the Thr75 site.

These mechanisms potentially underlying the discernibility of the DARPP-32 system need to be verified experimentally. If the experiments confirm our predictions, these dominant processes have implications for the vulnerability of the system and for possible therapeutic interventions in Parkinson's disease, schizophrenia, and drug addiction. For example, the DARPP-32 system is involved in the generation of dyskinesia, a severe disorder often associated with Parkinson's disease [41]. It will be of great potential impact to test whether interventions targeting these critical mechanisms in the DARPP-32 system can ameliorate this disorder. Efforts are currently in progress to develop drugs acting on medium spiny neurons [42-44], and it appears that our model and simulation results could facilitate a deeper and more detailed understanding of the specific effects of these drugs. By simulating the drug mechanism, such model analyses could investigate not only the effects on the anticipated target but also on all other processes that are affected within the DARPP-32 system. In addition, one could study with relative ease how such effects of the drug vary in different context of neuronal signals. Upon further testing and refinements, which will require additional data and information, the model will have the potential of assessing the effects of drug interventions on targets and processes located downstream of the DARPP-32 system.

## Methods

The DARPP-32 system includes various types of interactions, such as ligand-receptor binding, enzymatic reactions, activation, and inhibition. Our model, which accounts for all these components, is based on models developed by Lindskog and collaborators, the LeNovère's lab, and the Greengard's group [45-47], who studied various static and dynamic behaviors of the DARPP-32 system, but combines and extends these in a significant fashion. Its connectivity diagram is shown in Figures 1 and 2, and all mass action reactions are listed in Tables 2 and 3. A full, explicit and directly downloadable description of the model is available in Additional file 1.

**Table 2:** Reactions and rate constants of signal transduction for DARPP-32 phosphorylation in dendrites of medium spiny neurons in the striatum (see legend of Figure 1 for abbreviations)

Reaction	K_f_(nM^-1. ^s^-1^)^#^	K_b _(s^-1^)	K_c _(s^-1^)	**Ref**.
D1 + DA ↔ D1_DA	1.1E-3	10.0		[47]
D1_DA + G_αβγ _↔ D1_DA_G_αβγ_	6.0E-4	1.0E-3		[47]
D1 + G_αβγ _↔ D1_G_αβγ_	6.0E-5	3.0E-4		[47]
D1_G_αβγ_+ DA ↔ D1_DA_G_αβγ_	3.3E-3	10.0		[47]
D1_DA_G_αβγ _→ D1_DA + G_α_GTP+ G_βγ_	20.0^a^			[47]
G_α_GTP → G_α_GDP	10.0^a^			[47]
G_α_GDP + G_βγ _→ G_αβγ_	100.0			[47]
G_α_GTP + AC5 ↔ G_α_GTP_AC5	3.9E-2	50.0		[47]
G_α_GTP_AC5 + ATP ↔ G_α_GTP_AC5_ATP	1.3E-4	2.6E-1		[47]
G_α_GTP_AC5_ATP ↔ G_α_GTP_AC5 + cAMP	28.5^a^	2.6E-4^b^		[47]
PKA + 2 cAMP ↔ PKA_cAMP_2_	1.3E-5^c^	6.0E-3		[47]
PKA_cAMP_2 _+ 2 cAMP ↔ PKA_cAMP_4_	1.7E-5^c^	6.0E-2		[47]
PKA_cAMP_4 _↔ 2 PKAc + PKAr	5.1E-4^a^	4.8E-3^c^		[47]
PDE1 + cAMP ↔ PDE1_cAMP → PDE1 + AMP	2.0E-2	72.0	18.0	[47]
PDE4 + cAMP ↔ PDE4_cAMP → PDE4 + AMP	2.0E-2	72.0	18.0	[47]
PKAc + PDE1 ↔ PKAc_PDE1 → PKAc + PDE1p	6.0E-3	36.0	9.0	[48]
PDE1p → PDE1	1.0E-1^a^			[48]
PKAc + PDE4 ↔ PKAc_PDE4 → PKAc + PDE4p	6.0E-3	36.0	9.0	[48]
PDE4p → PDE4	1.0E-1^a^			[48]
→ Ca^2+^	2.5E+1^e^			[48]
Ca^2+^→	0.6^a^/1.7^a^			[48]
2 Ca^2+ ^+ PP2Bi ↔ PP2Bi_Ca_2_	1.0E-3^c^	1.0		[48]
2 Ca^2+ ^+ PP2Bi_Ca_2 _↔ PP2B	3.0E-3^c^	1.0		[48]
AC5 + Ca^2+ ^↔ AC5_Ca	1.0E-3	0.9		[47]
G_α_GTP + AC5_Ca ↔ G_α_GTP_AC5_Ca	1.9E-2	25.0		[47]
G_α_GTP_AC5_Ca + ATP ↔ G_α_GTP_AC5_Ca_ATP	6.0E-5	1.3E-1		[47]
G_α_GTP_AC5_Ca_ATP ↔ G_α_GTP_AC5_Ca + cAMP	14.2^a^	1.3E-4^b^		[47]
PP2A + 4 Ca^2+ ^↔ PP2Ac	7.7E-12^d^	1.0E-2		[47]
PP2A + PKAc ↔ PP2A_PKAc → PP2Ap + PKAc	2.5E-3	0.3	0.1	[49]
PP2Ap → PP2A	4.0E-3^a^			[47]
CK1 → CK1p	1.0^a^			[47]
PP2B + CK1p ↔ PP2B_CK1p → PP2B + CK1	3.0E-2	24.0	6.0	[47]
CDK5 + Ca^2+ ^↔ CDK5c	3.0E-3	1.0		

^a^: Unit in s^-1^; ^b^: Unit in nM^-1^·s^-1^; ^c^: Unit in nM^-2^·s^-1^; ^d^: Unit in nM^-4^·s^-1^; ^e^: Unit in nM·s^-1^

^#^: For a chemical reaction, K_f _is the rate constant for the forward process, K_b _is the rate constant for the backward process, while K_c _is the rate constant for the catalytic step in a Michaelis-Menten kinetics.

**Table 3:** Reactions and rate constants of DARPP-32 phosphorylation in dendrites of medium spiny neurons in the striatum (see legend of Figure 1 for abbreviations)

Reaction	K_f_(nM^-1^·s^-1^)^#^	K_b _(s^-1^)	K_c _(s^-1^)	**Ref**.
D + PKAc ↔ D_PKAc → D34 + PKAc	5.6E-3	10.8	2.7	[16,50]
D34 + PP2B ↔ D34_PP2B → D + PP2B	1.0E-3	2.0	0.5	[19]
D + CDK5 ↔ D_CDK5 → D75 + CDK5	4.5E-4	2.0	0.5	[47]
D75 + PP2Ap ↔ D75_PP2Ap → D + PP2Ap	4.0E-4	12.0	3.0	[47]
D75 + PP2A ↔ D75_PP2A → D + PP2A	1.0E-4	6.4	1.6	[47]
D75 + PP2Ac ↔ D75_PP2Ac → D + PP2Ac	4.0E-4	12.0	3.0	[47]
D + CK2 ↔ D_CK2 → D102 + CK2	4.0E-4	6.4	1.6	[51]
D102 → D	1.6^a^			
D + CK1 ↔ D_CK1 → D137 + CK1	4.4E-3	12.0	3.0	[25]
D137 + PP2C ↔ D137_PP2C → D + PP2C	7.5E-3	12.0	3.0	[47]
D102 + PKAc ↔ D102_PKAc → D34:102 + PKAc	5.6E-3	10.8	2.7	
D34:102 + PP2B ↔ D34:102_PP2B → D102 + PP2B	7.5E-5	0.12	0.03	
D137 + PKAc ↔ D137_PKAc → D34:137 + PKAc	5.6E-3	10.8	2.7	[16,50]
D34:137 + PP2B ↔ D34:137_PP2B → D137 + PP2B	7.5E-5	0.12	0.03	[25]
D102 + CDK5 ↔ D102_CDK5 → D75:102 + CDK5	4.5E-4	2.0	0.5	
D75:102 + PP2Ap ↔ D75:102_PP2Ap → D102 + PP2Ap	4.0E-4	12.0	3.0	
D75:102 + PP2A ↔ D75:102_PP2A → D102 + PP2A	1.0E-4	6.4	1.6	
D75:102 + PP2Ac ↔ D75:102_PP2Ac → D102 + PP2Ac	4.0E-4	12.0	3.0	
D137 + CDK5 ↔ D137_CDK5 → D75:137 + CDK5	4.5E-4	2.0	0.5	
D75:137 + PP2Ap ↔ D75:137_PP2Ap → D137 + PP2Ap	4.0E-4	12.0	3.0	
D75:137 + PP2A ↔ D75:137_PP2A → D137 + PP2A	1.0E-4	6.4	1.6	
D75:137 + PP2Ac ↔ D75:137_PP2Ac → D137 + PP2Ac	4.0E-4	12.0	3.0	
D34 + CK2 ↔ D34_CK2 → D34:102 + CK2	4.0E-4	6.4	1.6	
D34:102 → D34	1.6^a^			
D75 + CK2 ↔ D75_CK2 → D75:102 + CK2	4.0E-4	6.4	1.6	
D75:102 → D75	1.6^a^			
D34 + CK1 ↔ D34_CK1 → D34:137 + CK1	4.4E-3	12.0	3.0	[25]
D34:137 + PP2C ↔ D34:137_PP2C → D34 + PP2C	7.5E-3	12.0	3.0	[47]
D75 + CK1 ↔ D75_CK1 → D75:137 + CK1	4.4E-3	12.0	3.0	[25]
D75:137 + PP2C ↔ D75:137_PP2C → D75 + PP2C	7.5E-3	12.0	3.0	[47]
D102 + CK1 ↔ D102_CK1 → D102:137 + CK1	4.4E-3	12.0	3.0	
D102:137 + PP2C ↔ D102:137_PP2C → D102 + PP2C	7.5E-3	12.0	3.0	
D137 + CK2 ↔ D137_CK2 → D102:137 + CK2	4.0E-4	6.4	1.6	
D102:137 → D137	1.6^a^			
D34:102 + CK1 ↔ D34:102_CK1 → D34:102:137 + CK1	4.4E-3	12.0	3.0	
D34:102:137 + PP2C ↔ D34:102:137_PP2C → D34:102 + PP2C	7.5E-3	12.0	3.0	
D34:137 + CK2 ↔ D34:137_CK2 → D34:102:137 + CK2	4.0E-4	6.4	1.6	
D34:102:137 → D34:137	1.6^a^			
D102:137 + PKAc ↔ D102:137_PKAc → D34:102:137 + PKAc	5.6E-3	10.8	2.7	
D34:102:137 + PP2B ↔ D34:102:137_PP2B → D102:137 + PP2B	7.5E-5	0.12	0.03	
D75:102 + CK1 ↔ D75:102_CK1 → D75:102:137 + CK1	4.4E-3	12.0	3.0	
D75:102:137 + PP2C ↔ D75:102:137_PP2C → D75:102 + PP2C	7.5E-3	12.0	3.0	
D75:137 + CK2 ↔ D75:137_CK2 → D75:102:137 + CK2	4.0E-4	6.4	1.6	
D75:102:137 → D75:137	1.6^a^			
D102:137 + CDK5 ↔ D102:137_CDK5 → D75:102:137 + CDK5	4.5E-4	2.0	0.5	
D75:102:137 + PP2Ap ↔ D75:102:137_PP2Ap → D102:137 + PP2Ap	4.0E-4	12.0	3.0	
D75:102:137 + PP2A ↔ D75:102:137_PP2A → D102:137 + PP2A	1.0E-4	6.4	1.6	
D75:102:137 + PP2Ac ↔ D75:102:137_PP2Ac → D102:137 + PP2Ac	4.0E-4	12.0	3.0	
D34 + PP1 ↔ D34_PP1	0.4	0.6		[47]
D34_PP1 + PP2B ↔ D34_PP1_PP2B → D + PP1 + PP2B	1.0E-3	2.0	0.5	[47]
D34:102 + PP1 ↔ D34:102_PP1	0.4	0.6		
D34:102_PP1 + PP2B ↔ D34:102_PP1_PP2B → D102 + PP1 + PP2B	1.0E-3	2.0	0.5	
D34:137 + PP1 ↔ D34:137_PP1	0.4	0.6		
D34:137_PP1 + PP2B ↔ D34:137_PP1_PP2B → D137 + PP1 + PP2B	1.0E-3	2.0	0.5	
D34:102:137 + PP1 ↔ D34:102:137_PP1	0.4	0.6		
D34:102:137_PP1 + PP2B ↔ D34:102:137_PP1_PP2B → D102:137 + PP1 + PP2B	1.0E-3	2.0	0.5	
D75 + PKAc ↔ D75_PKAc	5.6E-3	10.8		[16]
D75:102 + PKAc ↔ D75:102_PKAc	5.6E-3	10.8		
D75:137 + PKAc ↔ D75:137_PKAc	5.6E-3	10.8		
D75:102:137 + PKAc ↔ D75:102:137_PKAc	5.6E-3	10.8		

^a^: Unit in s^-1^

^#^: For a chemical reaction, K_f _is the rate constant for the forward process, K_b _is the rate constant for the backward process, while K_c _is the rate constant for the catalytic step in a Michaelis-Menten kinetics.

Figure 1 indicates that dopamine can bind to its D1 receptors on the postsynaptic membrane, where it forms a complex with the corresponding G protein. The complex releases its alpha subunit, which binds to adenylate cyclase (AC5) and generates cAMP. This second messenger binds to the regulatory subunit of PKA and releases its catalytic subunit, which phosphorylates DARPP-32 at threonine 34 (Thr34, positioned according to the rat sequence). Counteracting this effect, the same site is dephosphorylated by the phosphatase PP2B. DARPP-32 can also be phosphorylated at a second threonine site (Thr75, positioned according to the rat sequence) by CDK5, a process which is counteracted by protein phosphatase 2A (PP2A), which mediates the release of the phosphate group from this site. DARPP-32 possesses two further phosphorylation sites, namely serine 102 and serine 137 (Ser102 and Ser137, positioned according to the rat sequence). Casein kinase 1 (CK1) and casein kinase 2 (CK2) execute the phosphorylation at Ser137 and Ser102, respectively.

These phosphorylation sites of DARPP-32 can be phosphorylated simultaneously, and transitions between different phosphorylation configurations are illustrated in Figure 2. However, phosphorylation activities at these sites are not independent of each other. For example, Ser102 and Ser137 phosphorylation of DARPP-32 can activate phosphorylation and repress dephosphorylation of DARPP-32 at Thr34, respectively. Importantly, DARPP-32 with Thr75 phosphorylation can bind to PKA and inhibit its capability to phosphorylate DARPP-32 at Thr34, which permits us to exclude the combined phosphorylation form of Thr34-Thr75 [16,47]. It is evident that the signal cascade becomes a complicated network when all possible interactions are taken into account.

We model this signaling system with ordinary differential equations for all variables (molecules) in Tables 2 and 3 according to the law of mass action. Each process is formulated as a product of concentrations of contributors raised to the relevant reaction orders (most of which are 1), and multiplied with the appropriate rate constant. An enzymatic reaction is assumed to follow a reversible Michaelis-Menten mechanism with forward, backward, and catalytic steps that are quantified by the rate constants *K*_*f*_, *K*_*b*_, and *K*_*c*_, respectively. A non-enzymatic reaction is treated with both forward and backward steps or as a single irreversible forward step reaction. The derivative of each variable is determined by the difference between the sum of its influxes and the sum of its effluxes; the equations are explicitly given in Additional file 1. The dynamics of the signaling network is obtained as the solution of the system of equations.

To gain quantitative insights, values for rate constants, reaction orders, and initial variable states are needed. This information is presented in Tables 2, 3 and 4, which were populated with information from the literature and databases such as BRENDA http://www.brenda-enzymes.info/. Some values were computed from kinetic data, while others (mostly rate constants for transitions between different forms of phosphorylation) were specified with reasonable estimates.

**Table 4:** Initial values for the DARPP-32 phosphorylation system in dendrites of medium spiny neurons in the striatum (see legend of Figure 1 for abbreviations)

Molecule	Concentration (nM)	Reference
DA	10.0	[45]
D1	5.0E+2	[45]
G_αβγ_	3.0E+3	[45]
AC5	2.5E+3	[45]
ATP	2.0E+6	[45]
PDE1	4.0E+3	[45]
PDE4	2.0E+3	[45]
DARPP-32	5.0E+4	[52]
PKA	1.2E+3	[53]
PP2Bi	4.0E+3	[45]
CDK5	1.8E+3	[45]
PP2A	2.0E+3	[54,55]
CK1	2.0E+3	
PP2C	2.0E+3	
PP1	5.0E+3	[56,57]
CK2	2.0E+3	

Once the numerical characterization was completed, it was straightforward to test different scenarios, for instance, by exposing the system to various signals and to random perturbations. Representative input patterns consist of combinations of the following signal specifications:

a. individual or combined dopamine and glutamate signals;

b. transient or sustained signals;

c. one-shot pulse or repetitive pulse signals;

d. signals of varying length;

e. signals of high or low amplitude.

The input signals were simulated in terms of different combinations of these specifications, and their effects on signal integration by DARPP-32 without or with perturbations were systematically evaluated.

The responses to the different input signals were examined from two distinct and relevant viewpoints. First, long-term effects indicate whether the system can switch to a different steady state, and if so, how large in magnitude the change is. Second, the transient effects, which are obtained as the dynamic behavior, reflect short-term responses of the system. Critical output variables for these responses are PKA and PP1. In addition to these two indicators, the phosphorylation profile of DARPP-32 can serve as a valuable sentinel, because it represents the internal state of the signal transduction system, while PKA and PP1 are functional proteins performing biochemical, cellular, and physiological regulations.

## Authors' contributions

ZQ carried out the modeling, analyses and interpretation of results, and manuscript writing. GWM participated in the design of the work and help to draft the manuscript. EOV conceived the study, interpreted results, and wrote the manuscript. All authors have read and approved the manuscript.

## Supplementary Material

Additional file 1 **The DARPP-32 model**. This is a computational model of the DARPP-32 system in the human striatum using ordinary differential equations. Click here for file
