# Supplementary material for: The internal state of medium spiny neurons varies in response to different input signals
Source: BMC Syst Biol. 2010 Mar 17;4:26. doi: 10.1186/1752-0509-4-26 (PMC2848196; doi:10.1186/1752-0509-4-26)
Supplement: Additional file 1 — The DARPP-32 model. This is a computational model of the DARPP-32 system in the human striatum using ordinary differential equations. [file 1752-0509-4-26-S1.DOC]

Supplements

**The Internal State of Medium Spiny Neurons Varies**

**in Response to Different Input Signals**

Zhen Qi, Gary W. Miller, Eberhard O. Voit

###### **Computational model of the DARPP-32 system in the human striatum**

We model the DARPP-32 system in the human striatum according to the law of mass action. Each process is formulated as a product of concentrations of contributors raised to the relevant reaction orders, and multiplied with the relevant rate constant. Our model is based on models developed by Lindskog’s, LeNovere’s, and Greengard’s groups [1-3]. While Lindskog’s model accounted for two phosphorylation sites of the DARPP-32 molecule; LeNovere’s model took into consideration three phosphorylation sites; and Greengard’s model used a random number for every rate constant, our model combined all these three models and extended them with additional processes from literature [4-6]. The result is given below in the format of the software PLAS, which can be downloaded for free from [7].

**PLAS implementation of the model of the DARPP-32 system**

X1' = 160 + 10 * X3 + 10 * X6 - 0.0011 * X2 * X1 - 0.0033 * X5 * X1 - 16 * X1

X2' = 10 * X3 + 0.0003 * X5 - 0.0011 * X2 * X1 - 0.00006 * X2 * X4

X3' = 0.0011 * X2 * X1 + 0.001 * X6 + 20 * X6 - 10 * X3 - 0.0006 * X3 * X4

X4' = 0.001 * X6 + 0.0003 * X5 + 100 * X9 * X8 - 0.0006 * X3 * X4 - 0.00006 * X2 * X4

X5' = 0.00006 * X2 * X4 + 10 * X6 - 0.0003 * X5 - 0.0033 * X5 * X1

X6' = 0.0006 * X3 * X4 + 0.0033 * X5 * X1 - 0.001 * X6 - 10 * X6 - 20 * X6

X7' = 20 * X6 + 50 * X11 + 25 * X32 - 10 * X7 - 0.039 * X7 * X10 - 0.019 * X7 * X31

X8' = 20 * X6 - 100 * X9 * X8

X9' = 10 * X7 - 100 * X9 * X8

X10' = 50 * X11 + 0.9 * X31 - 0.039 * X7 * X10 - 0.001 * X10 * X27

X11' = 0.039 * X7 * X10 + 0.26 * X12 + 28.5 * X12 - 50 * X11 - 0.00013 * X11 * 2000000 - 0.00026 * X11 * X14

X12' = 0.00013 * X11 * 2000000 + 0.00026 * X11 * X14 - 0.26 * X12 - 28.5 * X12

X13' = 0.006 * X15 - 0.000013 * X13 * X14^2.0

X14' = 28.5 * X12 + 2.0 * 0.006 * X15 + 2.0 * 0.06 * X16 + 72 * X20 + 72 * X22 + 14.2 * X33 - 0.00026 * X11 * X14 - 2.0 * 0.000013 * X13 * X14^2.0 - 2.0 * 0.000017 * X15 * X14^2.0 - 0.02 * X19 * X14 - 0.02 * X21 * X14 - 0.00013 * X32 * X14

X15' = 0.000013 * X13 * X14^2.0 + 0.06 * X16 - 0.006 * X15 - 0.000017 * X15 * X14^2.0

X16' = 0.000017 * X15 * X14^2.0 + 0.0048 * X17^2.0 * X18 - 0.06 * X16 - 0.00051 * X16

X17' = 2.0 * 0.00051 * X16 + 36 * X23 + 9 * X23 + 36 * X25 + 9 * X25 + 0.3 * X36 + 0.1 * X36 + 10.8 * X42 + 2.7 * X42 + 10.8 * X59 + 2.7 * X59 + 10.8 * X62 + 2.7 * X62 + 10.8 * X89 + 2.7 * X89 + 10.8 * X107 + 10.8 * X108 + 10.8 * X109 + 10.8 * X110 - 2.0 * 0.0048 * X17^2.0 * X18 - 0.006 * X17 * X19 - 0.006 * X17 * X21 - 0.0025 * X34 * X17 - 0.0056 * X41 * X17 - 0.0056 * X53 * X17 - 0.0056 * X55 * X17 - 0.0056 * X82 * X17 - 0.004 * X47 * X17 - 0.004 * X66 * X17 - 0.004 * X71 * X17 - 0.004 * X92 * X17

X18' = 0.00051 * X16 - 0.0048 * X17^2.0 * X18

X19' = 72 * X20 + 18 * X20 + 36 * X23 + 0.1 * X24 - 0.02 * X19 * X14 - 0.006 * X17 * X19

X20' = 0.02 * X19 * X14 - 72 * X20 - 18 * X20

X21' = 72 * X22 + 18 * X22 + 36 * X25 + 0.1 * X26 - 0.02 * X21 * X14 - 0.006 * X17 * X21

X22' = 0.02 * X21 * X14 - 72 * X22 - 18 * X22

X23' = 0.006 * X17 * X19 - 36 * X23 - 9 * X23

X24' = 9 * X23 - 0.1 * X24

X25' = 0.006 * X17 * X21 - 36 * X25 - 9 * X25

X26' = 9 * X25 - 0.1 * X26

X27' = 25 + 2.0 * 1 * X29 + 2.0 * 1 * X30 + 0.9 * X31 + 4.0 * 0.01 * X35 + 1 * X115 - 1.7 * X27 - 2.0 * 0.001 * X27^2.0 * X28 - 2.0 * 0.003 * X27^2.0 * X29 - 0.001 * X10 * X27 - 4.0 * 0.0000000000077 * X34 * X27^4.0 - 0.003 * X45 * X27

X28' = 1 * X29 - 0.001 * X27^2.0 * X28

X29' = 0.001 * X27^2.0 * X28 + 1 * X30 - 1 * X29 - 0.003 * X27^2.0 * X29

X30' = 0.003 * X27^2.0 * X29 + 24 * X40 + 6 * X40 + 2 * X44 + 0.5 * X44 + 0.12 * X61 + 0.03 * X61 + 0.12 * X64 + 0.03 * X64 + 0.12 * X90 + 0.03 * X90 + 2 * X100 + 0.5 * X100 + 2 * X102 + 0.5 * X102 + 2 * X104 + 0.5 * X104 + 2 * X106 + 0.5 * X106 - 1 * X30 - 0.03 * X30 * X39 - 0.001 * X43 * X30 - 0.000075 * X60 * X30 - 0.000075 * X63 * X30 - 0.000075 * X86 * X30 - 0.001 * X99 * X30 - 0.001 * X101 * X30 - 0.001 * X103 * X30 - 0.001 * X105 * X30

X31' = 0.001 * X10 * X27 + 25 * X32 - 0.9 * X31 - 0.019 * X7 * X31

X32' = 0.019 * X7 * X31 + 0.13 * X33 + 14.2 * X33 - 25 * X32 - 0.00006 * X32 * 2000000 - 0.00013 * X32 * X14

X33' = 0.00006 * X32 * 2000000 + 0.00013 * X32 * X14 - 0.13 * X33 - 14.2 * X33

X34' = 0.01 * X35 + 0.3 * X36 + 0.004 * X37 + 6.4 * X49 + 1.6 * X49 + 6.4 * X68 + 1.6 * X68 + 6.4 * X73 + 1.6 * X73 + 6.4 * X97 + 1.6 * X97 - 0.0000000000077 * X34 * X27^4.0 - 0.0025 * X34 * X17 - 0.0001 * X47 * X34 - 0.0001 * X66 * X34 - 0.0001 * X71 * X34 - 0.0001 * X92 * X34

X35' = 0.0000000000077 * X34 * X27^4.0 + 12 * X50 + 3 * X50 + 12 * X69 + 3 * X69 + 12 * X74 + 3 * X74 + 12 * X98 + 3 * X98 - 0.01 * X35 - 0.0004 * X47 * X35 - 0.0004 * X66 * X35 - 0.0004 * X71 * X35 - 0.0004 * X92 * X35

X36' = 0.0025 * X34 * X17 - 0.3 * X36 - 0.1 * X36

X37' = 0.1 * X36 + 12 * X48 + 3 * X48 + 12 * X67 + 3 * X67 + 12 * X72 + 3 * X72 + 12 * X96 + 3 * X96 - 0.004 * X37 - 0.0004 * X47 * X37 - 0.0004 * X66 * X37 - 0.0004 * X71 * X37 - 0.0004 * X92 * X37

X38' = 6 * X40 + 12 * X54 + 3 * X54 + 12 * X77 + 3 * X77 + 12 * X79 + 3 * X79 + 12 * X81 + 3 * X81 + 12 * X85 + 3 * X85 + 12 * X91 + 3 * X91 - 1 * X38 - 0.0044 * X41 * X38 - 0.0044 * X43 * X38 - 0.0044 * X47 * X38 - 0.0044 * X53 * X38 - 0.0044 * X60 * X38 - 0.0044 * X66 * X38

X39' = 1 * X38 + 24 * X40 - 0.03 * X30 * X39

X40' = 0.03 * X30 * X39 - 24 * X40 - 6 * X40

X41' = 10.8 * X42 + 0.5 * X44 + 2 * X46 + 3 * X48 + 1.6 * X49 + 3 * X50 + 6.4 * X52 + 1.6 * X53 + 12 * X54 + 3 * X57 + 0.5 * X100 + 4 * X111 - 0.0056 * X41 * X17 - 0.00045 * X41 * X45 - 0.0004 * X41 * X51 - 0.0044 * X41 * X38 - 0.0018 * X41 * X115

X42' = 0.0056 * X41 * X17 - 10.8 * X42 - 2.7 * X42

X43' = 2.7 * X42 + 2 * X44 + 6.4 * X75 + 1.6 * X60 + 12 * X77 + 3 * X78 + 0.6 * X99 - 0.001 * X43 * X30 - 0.0004 * X43 * X51 - 0.0044 * X43 * X38 - 0.4 * X43 * X58

X44' = 0.001 * X43 * X30 - 2 * X44 - 0.5 * X44

X45' = 2 * X46 + 0.5 * X46 + 2 * X65 + 0.5 * X65 + 2 * X70 + 0.5 * X70 + 2 * X95 + 0.5 * X95 + 1 * X115 - 0.00045 * X41 * X45 - 0.00045 * X53 * X45 - 0.00045 * X55 * X45 - 0.00045 * X82 * X45 - 0.003 * X45 * X27

X46' = 0.00045 * X41 * X45 - 2 * X46 - 0.5 * X46

X47' = 0.5 * X46 + 12 * X48 + 6.4 * X49 + 12 * X50 + 6.4 * X76 + 1.6 * X66 + 12 * X79 + 3 * X80 + 10.8 * X107 + 1 * X111 - 0.0004 * X47 * X37 - 0.0001 * X47 * X34 - 0.0004 * X47 * X35 - 0.0004 * X47 * X51 - 0.0044 * X47 * X38 - 0.004 * X47 * X17

X48' = 0.0004 * X47 * X37 - 12 * X48 - 3 * X48

X49' = 0.0001 * X47 * X34 - 6.4 * X49 - 1.6 * X49

X50' = 0.0004 * X47 * X35 - 12 * X50 - 3 * X50

X51' = 6.4 * X52 + 1.6 * X52 + 6.4 * X75 + 1.6 * X75 + 6.4 * X76 + 1.6 * X76 + 6.4 * X84 + 1.6 * X84 + 6.4 * X88 + 1.6 * X88 + 6.4 * X94 + 1.6 * X94 - 0.0004 * X41 * X51 - 0.0004 * X43 * X51 - 0.0004 * X47 * X51 - 0.0004 * X55 * X51 - 0.0004 * X63 * X51 - 0.0004 * X71 * X51

X52' = 0.0004 * X41 * X51 - 6.4 * X52 - 1.6 * X52

X53' = 1.6 * X52 + 10.8 * X59 + 0.03 * X61 + 2 * X65 + 3 * X67 + 1.6 * X68 + 3 * X69 + 12 * X81 + 3 * X83 + 0.5 * X102 + 4 * X112 - 1.6 * X53 - 0.0056 * X53 * X17 - 0.00045 * X53 * X45 - 0.0044 * X53 * X38 - 0.0018 * X53 * X115

X54' = 0.0044 * X41 * X38 - 12 * X54 - 3 * X54

X55' = 3 * X54 + 12 * X57 + 10.8 * X62 + 0.03 * X64 + 2 * X70 + 3 * X72 + 1.6 * X73 + 3 * X74 + 6.4 * X84 + 1.6 * X82 + 0.5 * X104 + 4 * X113 - 0.0075 * X55 * X56 - 0.0056 * X55 * X17 - 0.00045 * X55 * X45 - 0.0004 * X55 * X51 - 0.0018 * X55 * X115

X56' = 12 * X57 + 3 * X57 + 12 * X78 + 3 * X78 + 12 * X80 + 3 * X80 + 12 * X83 + 3 * X83 + 12 * X87 + 3 * X87 + 12 * X93 + 3 * X93 - 0.0075 * X55 * X56 - 0.0075 * X63 * X56 - 0.0075 * X71 * X56 - 0.0075 * X82 * X56 - 0.0075 * X86 * X56 - 0.0075 * X92 * X56

X57' = 0.0075 * X55 * X56 - 12 * X57 - 3 * X57

X58' = 0.6 * X99 + 0.5 * X100 + 0.6 * X101 + 0.5 * X102 + 0.6 * X103 + 0.5 * X104 + 0.6 * X105 + 0.5 * X106 - 0.4 * X43 * X58 - 0.4 * X60 * X58 - 0.4 * X63 * X58 - 0.4 * X86 * X58

X59' = 0.0056 * X53 * X17 - 10.8 * X59 - 2.7 * X59

X60' = 2.7 * X59 + 0.12 * X61 + 1.6 * X75 + 12 * X85 + 3 * X87 + 0.6 * X101 - 0.000075 * X60 * X30 - 1.6 * X60 - 0.0044 * X60 * X38 - 0.4 * X60 * X58

X61' = 0.000075 * X60 * X30 - 0.12 * X61 - 0.03 * X61

X62' = 0.0056 * X55 * X17 - 10.8 * X62 - 2.7 * X62

X63' = 2.7 * X62 + 0.12 * X64 + 3 * X77 + 12 * X78 + 6.4 * X88 + 1.6 * X86 + 0.6 * X103 - 0.000075 * X63 * X30 - 0.0075 * X63 * X56 - 0.0004 * X63 * X51 - 0.4 * X63 * X58

X64' = 0.000075 * X63 * X30 - 0.12 * X64 - 0.03 * X64

X65' = 0.00045 * X53 * X45 - 2 * X65 - 0.5 * X65

X66' = 0.5 * X65 + 12 * X67 + 6.4 * X68 + 12 * X69 + 1.6 * X76 + 12 * X91 + 3 * X93 + 10.8 * X108 + 1 * X112 - 0.0004 * X66 * X37 - 0.0001 * X66 * X34 - 0.0004 * X66 * X35 - 1.6 * X66 - 0.0044 * X66 * X38 - 0.004 * X66 * X17

X67' = 0.0004 * X66 * X37 - 12 * X67 - 3 * X67

X68' = 0.0001 * X66 * X34 - 6.4 * X68 - 1.6 * X68

X69' = 0.0004 * X66 * X35 - 12 * X69 - 3 * X69

X70' = 0.00045 * X55 * X45 - 2 * X70 - 0.5 * X70

X71' = 0.5 * X70 + 12 * X72 + 6.4 * X73 + 12 * X74 + 3 * X79 + 12 * X80 + 6.4 * X94 + 1.6 * X92 + 10.8 * X109 + 1 * X113 - 0.0004 * X71 * X37 - 0.0001 * X71 * X34- 0.0004 * X71 * X35 - 0.0075 * X71 * X56 - 0.0004 * X71 * X51 - 0.004 * X71 * X17

X72' = 0.0004 * X71 * X37 - 12 * X72 - 3 * X72

X73' = 0.0001 * X71 * X34 - 6.4 * X73 - 1.6 * X73

X74' = 0.0004 * X71 * X35 - 12 * X74 - 3 * X74

X75' = 0.0004 * X43 * X51 - 6.4 * X75 - 1.6 * X75

X76' = 0.0004 * X47 * X51 - 6.4 * X76 - 1.6 * X76

X77' = 0.0044 * X43 * X38 - 12 * X77 - 3 * X77

X78' = 0.0075 * X63 * X56 - 12 * X78 - 3 * X78

X79' = 0.0044 * X47 * X38 - 12 * X79 - 3 * X79

X80' = 0.0075 * X71 * X56 - 12 * X80 - 3 * X80

X81' = 0.0044 * X53 * X38 - 12 * X81 - 3 * X81

X82' = 3 * X81 + 12 * X83 + 1.6 * X84 + 10.8 * X89 + 0.03 * X90 + 2 * X95 + 3 * X96 + 1.6 * X97 + 3 * X98 + 0.5 * X106 + 4 * X114 - 0.0075 * X82 * X56 - 1.6 * X82 - 0.0056 * X82 * X17 - 0.00045 * X82 * X45 - 0.0018 * X82 * X115

X83' = 0.0075 * X82 * X56 - 12 * X83 - 3 * X83

X84' = 0.0004 * X55 * X51 - 6.4 * X84 - 1.6 * X84

X85' = 0.0044 * X60 * X38 - 12 * X85 - 3 * X85

X86' = 3 * X85 + 12 * X87 + 1.6 * X88 + 2.7 * X89 + 0.12 * X90 + 0.6 * X105 - 0.0075 * X86 * X56 - 1.6 * X86 - 0.000075 * X86 * X30 - 0.4 * X86 * X58

X87' = 0.0075 * X86 * X56 - 12 * X87 - 3 * X87

X88' = 0.0004 * X63 * X51 - 6.4 * X88 - 1.6 * X88

X89' = 0.0056 * X82 * X17 - 10.8 * X89 - 2.7 * X89

X90' = 0.000075 * X86 * X30 - 0.12 * X90 - 0.03 * X90

X91' = 0.0044 * X66 * X38 - 12 * X91 - 3 * X91

X92' = 3 * X91 + 12 * X93 + 1.6 * X94 + 0.5 * X95 + 12 * X96 + 6.4 * X97 + 12 * X98 + 10.8 * X110 + 1 * X114 - 0.0075 * X92 * X56 - 1.6 * X92 - 0.0004 * X92 * X37 - 0.0001 * X92 * X34 - 0.0004 * X92 * X35 - 0.004 * X92 * X17

X93' = 0.0075 * X92 * X56 - 12 * X93 - 3 * X93

X94' = 0.0004 * X71 * X51 - 6.4 * X94 - 1.6 * X94

X95' = 0.00045 * X82 * X45 - 2 * X95 - 0.5 * X95

X96' = 0.0004 * X92 * X37 - 12 * X96 - 3 * X96

X97' = 0.0001 * X92 * X34 - 6.4 * X97 - 1.6 * X97

X98' = 0.0004 * X92 * X35 - 12 * X98 - 3 * X98

X99' = 0.4 * X43 * X58 + 2 * X100 - 0.6 * X99 - 0.001 * X99 * X30

X100' = 0.001 * X99 * X30 - 2 * X100 - 0.5 * X100

X101' = 0.4 * X60 * X58 + 2 * X102 - 0.6 * X101 - 0.001 * X101 * X30

X102' = 0.001 * X101 * X30 - 2 * X102 - 0.5 * X102

X103' = 0.4 * X63 * X58 + 2 * X104 - 0.6 * X103 - 0.001 * X103 * X30

X104' = 0.001 * X103 * X30 - 2 * X104 - 0.5 * X104

X105' = 0.4 * X86 * X58 + 2 * X106 - 0.6 * X105 - 0.001 * X105 * X30

X106' = 0.001 * X105 * X30 - 2 * X106 - 0.5 * X106

X107' = 0.004 * X47 * X17 - 10.8 * X107

X108' = 0.004 * X66 * X17 - 10.8 * X108

X109' = 0.004 * X71 * X17 - 10.8 * X109

X110' = 0.004 * X92 * X17 - 10.8 * X110

X111' = 0.0018 * X41 * X115 - 4 * X111 - 1 * X111

X112' = 0.0018 * X53 * X115 - 4 * X112 - 1 * X112

X113' = 0.0018 * X55 * X115 - 4 * X113 - 1 * X113

X114' = 0.0018 * X82 * X115 - 4 * X114 - 1 * X114

X115' = 0.003 * X45 * X27 + 4 * X111 + 1 * X111 + 4 * X112 + 1 * X112 + 4 * X113 + 1 * X113 + 4 * X114 + 1 * X114 - 1 * X115 - 0.0018 * X41 * X115 - 0.0018 * X53 * X115 - 0.0018 * X55 * X115 - 0.0018 * X82 * X115

X1 = 10

X2 = 500

X3 = 0.000001

X4 = 3000

X5 = 0.000001

X6 = 0.000001

X7 = 0.000001

X8 = 0.000001

X9 = 0.000001

X10 = 2500

X11 = 0.000001

X12 = 0.000001

X13 = 1200

X14 = 0.000001

X15 = 0.000001

X16 = 0.000001

X17 = 0.000001

X18 = 0.000001

X19 = 4000

X20 = 0.000001

X21 = 2000

X22 = 0.000001

X23 = 0.000001

X24 = 0.000001

X25 = 0.000001

X26 = 0.000001

X27 = 0.000001

X28 = 4000

X29 = 0.000001

X30 = 0.000001

X31 = 0.000001

X32 = 0.000001

X33 = 0.000001

X34 = 2000

X35 = 0.000001

X36 = 0.000001

X37 = 0.000001

X38 = 2000

X39 = 0.000001

X40 = 0.000001

X41 = 50000

X42 = 0.000001

X43 = 0.000001

X44 = 0.000001

X45 = 1800

X46 = 0.000001

X47 = 0.000001

X48 = 0.000001

X49 = 0.000001

X50 = 0.000001

X51 = 2000

X52 = 0.000001

X53 = 0.000001

X54 = 0.000001

X55 = 0.000001

X56 = 2000

X57 = 0.000001

X58 = 5000

X59 = 0.000001

X60 = 0.000001

X61 = 0.000001

X62 = 0.000001

X63 = 0.000001

X64 = 0.000001

X65 = 0.000001

X66 = 0.000001

X67 = 0.000001

X68 = 0.000001

X69 = 0.000001

X70 = 0.000001

X71 = 0.000001

X72 = 0.000001

X73 = 0.000001

X74 = 0.000001

X75 = 0.000001

X76 = 0.000001

X77 = 0.000001

X78 = 0.000001

X79 = 0.000001

X80 = 0.000001

X81 = 0.000001

X82 = 0.000001

X83 = 0.000001

X84 = 0.000001

X85 = 0.000001

X86 = 0.000001

X87 = 0.000001

X88 = 0.000001

X89 = 0.000001

X90 = 0.000001

X91 = 0.000001

X92 = 0.000001

X93 = 0.000001

X94 = 0.000001

X95 = 0.000001

X96 = 0.000001

X97 = 0.000001

X98 = 0.000001

X99 = 0.000001

X100 = 0.000001

X101 = 0.000001

X102 = 0.000001

X103 = 0.000001

X104 = 0.000001

X105 = 0.000001

X106 = 0.000001

X107 = 0.000001

X108 = 0.000001

X109 = 0.000001

X110 = 0.000001

X111 = 0.000001

X112 = 0.000001

X113 = 0.000001

X114 = 0.000001

X115 = 0.000001

t0 = 0

tf = 3000

hr = 1

**Table A1. Variable names in equations and their molecular entities**

| **Variable** | Molecular entity | **Abbreviation** |
| --- | --- | --- |
| *X1* | Dopamine | DA |
| *X2* | Dopamine D1 receptor | D1 |
| *X3* | The complex of dopamine and dopamine D1 receptor | DA_D1 |
| *X4* | G protein | Gαβγ |
| *X5* | The complex of G protein and dopamine D1 receptor | D1_ Gαβγ |
| *X6* | The complex of dopamine, dopamine D1 receptor, and G protein | D1_DA_Gαβγ |
| *X7* | α subunit of G protein with GTP | GαGTP |
| *X8* | β and γ subunits of G protein | Gβγ |
| *X9* | α subunit of G protein with GDP | GαGDP |
| *X10* | Adenylate cyclase | AC5 |
| *X11* | The complex of GαGTP and AC5 | GαGTP_AC5 |
| *X12* | The complex of GαGTP, AC5, and ATP | GαGTP_AC5_ATP |
| *X13* | Protein kinase A | PKA |
| *X14* | Cyclic AMP | cAMP |
| *X15* | The complex of PKA and two cAMP | PKA_cAMP_2_ |
| *X16* | The complex of PKA and four cAMP | PKA_cAMP_4_ |
| *X17* | The catalytic subunit of PKA | PKAc |
| *X18* | The regulatory subunit of PKA | PKAr |
| *X19* | Phosphodiesterase 1 | PDE1 |
| *X20* | The complex of PDE1 and cAMP | PDE1_cAMP |
| *X21* | Phosphodiesterase 4 | PDE4 |
| *X22* | The complex of PDE4 and cAMP | PDE4_cAMP |
| *X23* | The complex of PKAc and PDE1 | PKAc_PDE1 |
| *X24* | Phosphorylated PDE1 | PDE1p |
| *X25* | The complex of PKAc and PDE4 | PKAc_PDE4 |
| *X26* | Phosphorylated PDE4 | PDE4p |
| *X27* | Calcium cation | Ca2+ |
| *X28* | Protein phosphatase 2B | PP2Bi |
| *X29* | The complex of PP2Bi and two calcium cations | PP2Bi_Ca2 |
| *X30* | Activated protein phosphatase 2B | PP2B |
| *X31* | The complex of AC5 and calcium cation | AC5_Ca |
| *X32* | The complex of GαGTP, AC5, and calcium cation | GαGTP_AC5_Ca |
| *X33* | The complex of GαGTP, AC5, calcium cation, ATP | GαGTP_AC5_Ca_ATP |
| *X34* | Protein phosphatase 2A | PP2A |
| *X35* | Activated PP2A | PP2Ac |
| *X36* | The complex of PP2A and PKAc | PP2A_PKAc |
| *X37* | Phosphorylated PP2A | PP2Ap |
| *X38* | Casein kinase 1 | CK1 |
| *X39* | Phosphorylated CK1 | CK1p |
| *X40* | The complex of PP2B and CK1 | PP2B_CK1p |
| *X41* | Unphosphorylated DARPP-32 | D |
| *X42* | The complex of DARPP-32 and PKAc | D_PKAc |
| *X43* | DARPP-32 with phosphorylation at threonine 34 | D34 |
| *X44* | The complex of D34 and PP2B | D34_PP2B |
| *X45* | Cyclin-dependent kinase 5 | CDK5 |
| *X46* | The complex of DARPP-32 and CDK5 | D_CDK5 |
| *X47* | DARPP-32 with phosphorylation at threonine 75 | D75 |
| *X48* | The complex of D75 and PP2Ap | D75_PP2Ap |
| *X49* | The complex of D75 and PP2A | D75_PP2A |
| *X50* | The complex of D75 and PP2Ac | D75_PP2Ac |
| *X51* | Casein kinase 2 | CK2 |
| *X52* | The complex of DARPP-32 and CK2 | D_CK2 |
| *X53* | DARPP-32 with phosphorylation at serine 102 | D102 |
| *X54* | The complex of DARPP-32 and CK1 | D_CK1 |
| *X55* | DARPP-32 with phosphorylation at serine 137 | D137 |
| *X56* | Protein phosphatase 2C | PP2C |
| *X57* | The complex of D137 and PP2C | D137_PP2C |
| *X58* | Protein phosphatase-1 | PP1 |
| *X59* | The complex of D102 and PKAc | D102_PKAc |
| *X60* | DARPP-32 with phosphorylation at sites 34 and 102 | D34:102 |
| *X61* | The complex of D34:102 and PP2B | D34:102_PP2B |
| *X62* | The complex of D137 and PKAc | D137_PKAc |
| *X63* | DARPP-32 with phosphorylation at sites 34 and 137 | D34:137 |
| *X64* | The complex of D34:137 and PP2B | D34:137_PP2B |
| *X65* | The complex of D102 and CDK5 | D102_CDK5 |
| *X66* | DARPP-32 with phosphorylation at sites 75 and 102 | D75:102 |
| *X67* | The complex of D75:102 and PP2Ap | D75:102_PP2Ap |
| *X68* | The complex of D75:102 and PP2A | D75:102_PP2A |
| *X69* | The complex of D75:102 and PP2Ac | D75:102_PP2Ac |
| *X70* | The complex of D137 and CDK5 | D137_CDK5 |
| *X71* | DARPP-32 with phosphorylation at sites 75 and 137 | D75:137 |
| *X72* | The complex of D75:137 and PP2Ap | D75:137_PP2Ap |
| *X73* | The complex of D75:137 and PP2A | D75:137_PP2A |
| *X74* | The complex of D75:137 and PP2Ac | D75:137_PP2Ac |
| *X75* | The complex of D34 and CK2 | D34_CK2 |
| *X76* | The complex of D75 and CK2 | D75_CK2 |
| *X77* | The complex of D34 and CK1 | D34_CK1 |
| *X78* | The complex of D34:137 and PP2C | D34:137_PP2C |
| *X79* | The complex of D75 and CK1 | D75_CK1 |
| *X80* | The complex of D75:137 and PP2C | D75:137_PP2C |
| *X81* | The complex of D102 and CK1 | D102_CK1 |
| *X82* | DARPP-32 with phosphorylation at sites 102 and 137 | D102:137 |
| *X83* | The complex of D102:137 and PP2C | D102:137_PP2C |
| *X84* | The complex of D137 and CK2 | D137_CK2 |
| *X85* | The complex of D34:102 and CK1 | D34:102_CK1 |
| *X86* | DARPP-32 with phosphorylation at sites 34, 102 and 137 | D34:102:137 |
| *X87* | The complex of D34:102:137 and PP2C | D34:102:137_PP2C |
| *X88* | The complex of D34:137 and CK2 | D34:137_CK2 |
| *X89* | The complex of D102:137 and PKAc | D102:137_PKAc |
| *X90* | The complex of D34:102:137 and PP2B | D34:102:137_PP2B |
| *X91* | The complex of D75:102 and CK1 | D75:102_CK1 |
| *X92* | DARPP-32 with phosphorylation at sites 75, 102 and 137 | D75:102:137 |
| *X93* | The complex of D75:102:137 and PP2C | D75:102:137_PP2C |
| *X94* | The complex of D75:137 and CK2 | D75:137_CK2 |
| *X95* | The complex of D102:137 and CDK5 | D102:137_CDK5 |
| *X96* | The complex of D75:102:137 and PP2Ap | D75:102:137_PP2Ap |
| *X97* | The complex of D75:102:137 and PP2A | D75:102:137_PP2A |
| *X98* | The complex of D75:102:137 and PP2Ac | D75:102:137_PP2Ac |
| *X99* | The complex of D34 and PP1 | D34_PP1 |
| *X100* | The complex of D34, PP1, and PP2B | D34_PP1_PP2B |
| *X101* | The complex of D34:102 and PP1 | D34:102_PP1 |
| *X102* | The complex of D34:102, PP1, and PP2B | D34:102_PP1_PP2B |
| *X103* | The complex of D34:137 and PP1 | D34:137_PP1 |
| *X104* | The complex of D34:137, PP1, and PP2B | D34:137_PP1_PP2B |
| *X105* | The complex of D34:102:137 and PP1 | D34:102:137_PP1 |
| *X106* | The complex of D34:102:137, PP1, and PP2B | D34:102:137_PP1_PP2B |
| *X107* | The complex of D75 and PKAc | D75_PKAc |
| *X108* | The complex of D75:102 and PKAc | D75:102_PKAc |
| *X109* | The complex of D75:137 and PKAc | D75:137_PKAc |
| *X110* | The complex of D75:102:137 and PKAc | D75:102:137_PKAc |
| *X111* | The complex of DARPP-32 and CDK5c | D_CDK5c |
| *X112* | The complex of D102 and CDK5c | D102_CDK5c |
| *X113* | The complex of D137 and CDK5c | D137_CDK5c |
| *X114* | The complex of D102:137 and CDK5c | D102:137_CDK5c |
| *X115* | Activated CDK5 | CDK5c |

***References***
